# 

**Published:** 1891-06-06

**Authors:** 


PRICE ONE PENNY.

No. 245, Vol. X.—June 6th, 1891.

[Registered at the General Post Office as a Newspaper for transmission in the United Kingdom and Abroad.]

WITH EXTRA NURSING SUPPLEMENT.
Per Annum, 6s. 6d.; post free.
Monthly Parts, 6d.; post free 8d.
Ditto per Annum, 8s., post free.

### THE HOSPITAL

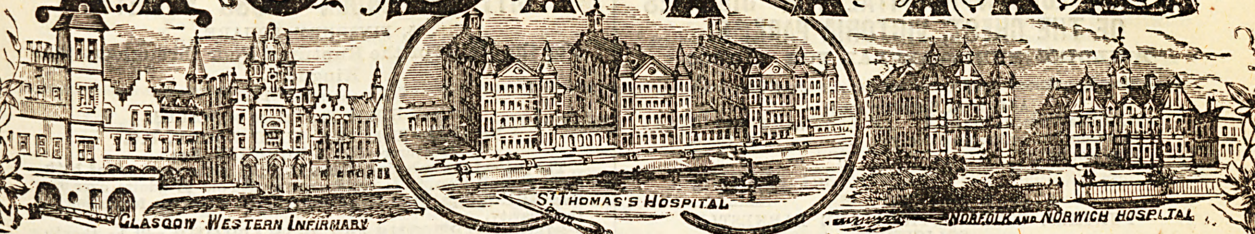

A WEEKLY JOURNAL OF

### Science, Medicine, Nursing and Philanthropy.

SATURDAY, JUNE 6th, 1891.

Results - - - - - - - - - 109

Passing Topics.—The R.B.N.A. at Bay - - - - 110

The Doctor's Armchair.—Derby and Oxford - - - 111

Everybody's Page - - - - - - - - 112

Editor's Letter Box.—The Influenza - - - - 112

The Teaching of Hygiene in Board Schools - - 113

Notes and News - - - - - - - - 114

General Charities—Scraps and Gleanings - - - 115

Annotations.—The Laureate and the Nurses—Vegetarianism and Influenza—Personal Troubles - - - - - - 116

Neuritis of the Peripheral Nerves, III.—By J. Michell Clarke, M.A., M.B., M.R.C.P. - - - - 119

The Practitioners' Mirror and Retrospect—

SURGERY.—The Surgery of Joints - - - - - - 117

THERAPEUTICS.—Eugenia Jambolana in Diabetes Mellitus 117

THE PRACTITIONER'S BOOKSHELF.—The Monthly Medical Review - - - - - - - - - 119

NEW DRUGS, APPLIANCES, AND THINGS MEDICAL.—The "Half-minute" Clinical Thermometer—Plasters and Bandages - - - - - - - - - 119

Portals of Health - - - - - - - - 120

The Student of Medicine.—The United Hospitals Athletic Club—Appointments—Vacancies—Notes from the Schools—Scholarships and Prizes—Athletics.

EXTRA SUPPLEMENT—THE NURSING MIRROR.

En Passant.—Nottingham Nurses—The Dewsbury Inquest—Upper Norwood District Nursing—Short Items—Nurses' Missionary Association—Another Home—To Would be Nurses—The Belfast Society - - - - - - - liii

Lectures on Surgical Ward Work and Nursing.—By ALEX. MILES, M.B. Edin., C.M., F.R.C.S.E.—Lecture XXVI.—A Box Splint Gooch Splint—Thomas' Splints - liv

The Queen at Derby - - - - - - - - liv

Appointments - - - - - - - - - lv

For Reading to the Sick—Work in Idleness - - - - lv

A Year in a German Hospital - - - - - - - lvi

The Royal British Nurses' Association and its Register - - - - - - - - - - lvii

Everybody's Opinion.—The Dulness of Domestic Service lvii

Wants and Workers—Notes and Queries - - - lvii

Off Duty.—"Into the Haven"—Presentations - - - lviii

Princess Louise Augusta's Wedding Present - lviii

Where to Go—Amusements and Relaxation - - lviii

